# Expansion Properties and Diffusion of Blowing Agent for Vinylidene Chloride Copolymer Thermally Expandable Microspheres

**DOI:** 10.3390/ma13173673

**Published:** 2020-08-20

**Authors:** Guiming Xie, Zhiyang Wang, Yongzhong Bao

**Affiliations:** 1 School of Chemistry and Chemical Engineering, Guizhou University, Guiyang 550025, China; wangzhiiyaa@126.com; 2 State Key Laboratory of Chemical Engineering, College of Chemical and Biological Engineering, Zhejiang University, Hangzhou 310027, China

**Keywords:** vinylidene chloride copolymer, thermally expandable microspheres, blowing agent, diffusion, expansion properties

## Abstract

Vinylidene chloride copolymer microspheres were synthesized by in situ suspension copolymerization of vinylidene chloride (VDC), methyl methacrylate (MMA), and/or acrylonitrile (AN) in the presence of a paraffin blowing agent. The effects of shell polymer properties including compositions, glass transition temperature (*T*_g_), crosslinking degree, blowing agent type, and encapsulation ratio (*E*_r_) on the expansion properties of copolymer microspheres were investigated. Moreover, the diffusion properties of blowing agent in copolymer microspheres were studied. The results show that VDC-MMA-AN copolymer microspheres exhibited excellent expansion properties, and the volume expansion ratio (*E*_v_) and the apparent density were decreased over 40 times, but it was difficult to expand for the VDC-MMA copolymer microspheres. In addition, the moderately crosslinked inside of the polymer shell enhanced the *E*_v_ more than 30 and the stable expansion temperature range (*T*_r_) was about 30 °C by adding 0.2–0.4 wt% of divinyl benzene. The *T*_g_ of the shell polymer must be higher than the boiling point of the blowing agent as a prerequisite; the lower the boiling point of the blowing agent, the higher the internal gas pressure driven microsphere expansion, and the wider the *T*_r_. By increasing the *E*_r_ of blowing agent improved the *E*_v_ of the microspheres. The diffusion of pentane blowing agent in VDC-MMA-AN copolymer microspheres were divided into Fick diffusion and non-Fick diffusion.

## 1. Introduction

Thermally expandable microsphere is a polymer particle with blowing agent (low boiling point liquid paraffin or other compounds) as a core and thermoplastic polymer as a shell. It can be dramatically expanded when it was heated higher than the boiling point of blowing agent and the polymer softening temperature. After expansion, a lower-density expanded microsphere was obtained [1]. The density can be reduced from about 1100 kg·m^−3^ to approximately 30 kg·m^−3^ [2,3]. Thermally expandable microspheres are widely used in packaging, insulation, coating, decoration, etc., which can reduce the weight of products and save amount of main materials. Moreover, they have been used for making a variety of foaming materials as well. Generally, a foaming agent in plastic or rubber, not only reduces the product density, but also endows products with sound insulation or heat insulation [4,5,6,7]. When they were added to coating, printing ink, the three-dimensional effect was generated or the surface characteristics of products was strengthened [8]. When they are applied in paper, paperboard, or non-woven fabric, the stereo effect of the products was formed and their anti-slip effect was enhanced [9]. Thermally expandable microspheres not only can be pre-foamed and then added to inks and coatings, but also can be mixed with plastics and rubber in the form of foaming in the process of processing [1,4,5,6,7,8,10,11,12]. Additionally, they were employed as a sacrificial template to fabricate macro-porous ceramic materials via a gel-casting process [13]. Used to modify structural adhesives to increase the joint strength by creating an adhesive functionally modified along the overlap of the joint by gradual heating and/or to heal the adhesive in case of damage [14,15,16]. When the surface of microspheres are functionalized, they are used to fabricate conductive thermally expandable microspheres [17,18]. Besides, thermally expandable microspheres were applied as single-use pumps or valves in microfluidic systems [19,20].

The expansion properties are the important basis for determining the application effect of thermally expandable microspheres and irrelevant with their form. There are many factors that affect the expansion properties of thermally expandable microspheres, including the composition of shell polymer, *T*_g_, viscoelasticity, and mechanical properties in the expansion process under the high temperature conditions, the type and encapsulated content of blowing agent, and the foaming technology, etc. In addition, adding crosslinking agent will increase the materials strength of the shell polymer under the condition of high viscoelasticity or viscous state and then increased the heat resistance and expansion properties of microspheres.

A great number of patents and articles have been reported related to the influence of composition and viscoelasticity of shell polymer and the type of blowing agent on the expansion properties of thermally expandable microspheres. For example, Kawaguchi et al. have studied the relationship between crosslinking density or viscoelasticity of AN-based copolymers and microspheres expansion properties [21,22]. They found out crosslinking and controlling the rheological properties of the shell polymer played a very critical role during expansion, an optimally crosslinked polymer benefitted to expand, without collapsing and rupturing of microspheres. However, excessive crosslinking restricted expansion, whereas insufficient crosslinking resulted in expanded microspheres collapse and rupture. They concluded that the modification of microspheres shell properties by crosslinking had a trade-off relationship between shrinkage protection and expandability enhancement. They also studied the influence of monomer chemical structure on heat resistance of thermally expandable microspheres [23]. Jonsson et al. have studied the effects of blowing agent structure, content and crosslinking agent structure on expansion properties of poly (AN-co-methacrylonitrile) microspheres [24,25,26]. The structure and physical properties (i.e., the boiling point and vapor pressure) of the blowing agent had a significant influence on the expansion properties of the microspheres. The blowing agent with a branch chain was not easy to spread out of the microspheres, and maintained sufficient pressure to sustain the outstanding expansion behavior. They also found the crosslinking agent of the 1,4-butanediol dimethacrylate improved the onset temperature of expansion of the microspheres. Our previous research [27] has investigated the effects of the monomer composition, crosslinking agent content, blowing agent type, and content on the morphology and blowing agent encapsulation efficiency of the microspheres, and found out the porous and non-core–shell structure microspheres with a low encapsulation efficiency of blowing agent were formed when VDC was copolymerized with individual AN or MMA, whereas microspheres with a core–shell structure and a high encapsulation efficiency of blowing agent were formed when the feeding monomer mass ratios of VDC–AN–MMA were 50/20/30, 60/20/20, and 70/20/10. Addition of 0.2–0.4 wt% divinyl benzene crosslinking agent facilitated the formation of the core–shell structure microspheres with a high encapsulation efficiency of blowing agent. Microspheres with wrinkled surfaces and a pronounced internal topography were formed as the carbon atom number of isopentane, pentane, hexane, and heptane increased from 5 to 7, but it was favorable to improvement of encapsulation efficiency of blowing agent. However, further studies have found that microspheres with a core-shell structure or a high encapsulation efficiency of blowing agent were the necessary conditions for microspheres expansion. But it does not mean that there must be a splendid expansion property. Because the expansion properties of microspheres are determined by *T*_g_, viscoelastic properties and internal gas pressure generated by the blowing agent during the expansion process. When they are matched properly, microspheres will have excellent expansion properties. However, the systematic relationship among the polymer properties, blowing agent, and the expansion properties of thermally expandable microspheres has not been established.

The encapsulated content of the blowing agent is an important factor in determining the expansion properties of microspheres. The barrier performance of the shell polymer of the microspheres is crucial in preventing it from spreading outward. Otherwise, the escaping of blowing agent will not provide enough internal gas pressure to drive the microspheres expansion. Kawaguchi et al. have proposed that the polymer shell layer barrier performance can be characterized by the density of the cohesion [23]. The homopolymers formed by AN or VDC are semi-crystalline polymers with excellent barrier properties. Therefore, VDC and AN are particularly used as the primary monomers for the preparation of low-temperature thermally expandable microspheres [3,28]. In this study, VDC, MMA, or/and AN as the monomers were used for obtaining the low-temperature thermally expandable copolymer microspheres having low expansion start.

The encapsulated content of blowing agent in microspheres is a key factor in the expansion properties of thermally expandable microspheres. Low boiling point blowing agents, especially in low-temperature thermally expandable microspheres, are easily dissipated during storage and transport by the permeation diffusion or volatilization through micropore of the shell polymer. Therefore, it is of great significance to study the diffusion characteristics of blowing agent in polymer microspheres for selecting suitable storage conditions of thermally expandable microspheres. In this paper, the effects of the composition, *T*_g_, crosslinking degree, blowing agent type, and *E*_r_ on the expansion properties of vinylidene chloride copolymer thermally expandable microspheres were studied. The diffusion properties of blowing agent in VDC-MMA-AN copolymer microspheres were investigated, and the storage and transport conditions of low-temperature vinylidene chloride copolymer thermally expandable microspheres were explored.

## 2. Materials and Methods

### 2.1. Materials

Vinylidene chloride (VDC, polymerization grade) was supplied by Juhua Group Co. (Hangzhou, China) and distilled twice in an atmosphere of N_2_. Acrylonitrile (AN, J&K Scientific Ltd., Beijing, China) and methyl methacrylate (MMA, Lingfeng Chemical, Shanghai, China) were distilled at reduced pressures. Divinyl benzene (DVB; m- and p-mixture, J&K Scientific Ltd., Beijing, China), 2,2′-azodiisobutyronitrile (AIBN), isopentane, pentane, hexane, heptanes, *N, N*-dimethylformamide, methanol, potassium dichromate, citric acid, and sodium chloride were chemically pure reagents and were purchased from Lingfeng Chemical (Shanghai, China). Hydroxypropyl methylcellulose and methylcellulose were supplied by Dow Chemical Co. (Midland, MI, USA). The deionized water was used for all experiments.

### 2.2. Preparation of Vinylidene Chloride Copolymer Thermally Expandable Microspheres

In a typical experiment, polymerizations were performed according to the general procedure described in our previous research. The morphologies of copolymer microspheres can also be seen in our previous research.Total of 300 mL of deionized water, 50 mL of hydroxypropyl methylcellulose dispersant aqueous solution (2 wt%), 100 mL of methylcellulose aqueous solution (1 wt%), and water-phase additives (4 wt% sodium chloride, 2.5 wt% citric acid, and 0.1 wt% potassium dichromate on the basis of the monomer content) were mixed to obtain the water phase. Amounts of 50 g of VDC, 20 g of AN, 30 g of MMA, 0.2 g of DVB, 2 g of AIBN, and 30 g of pentane were added to a beaker placed in an ice-water bath and mixed to obtain the oil phase. The previous water phase and oil phases were added to the autoclave, and air was removed from the upper part of the reactor by the purging of nitrogen to about 4 MPa and three release cycles. The mixture in the reactor was agitated for 30 min at a rate of 1200 rpm and at room temperature. Then, the agitation rate was deceased to 500 rpm, and the temperature of the reactants was raised to 60 °C to start the polymerization. The reaction proceeded for 15 h and was terminated by cooling to room temperature. The products were separated and dried below 40 °C [27].

### 2.3. Characterization

Determination of blowing agent encapsulated content: the thermogravimetric curve of the copolymer microspheres was recorded with a Pyris 1 thermogravimetric analyzer (TGA, PerkinElmer Co., Waltham, MA, USA). The sample was heated from 50 °C to 650 °C at a heating rate of 20 °C/min under an N_2_ atmosphere. A polymer sample with no encapsulated blowing agent was used to determine the amount of residual water and monomers. Thus, the content of encapsulated blowing agent in the microspheres was calculated from the thermogravimetric analysis results after the elimination of the residual water and monomer (ca. 2.4%). The *E*_r_ of the blowing agent was defined as the ratio between the weight of the blowing agent encapsulated in the microspheres and the weight of the microspheres [27].

Determination of the composition of shell polymer of VDC-MMA-AN copolymer microspheres: the microspheres samples were extracted with methanol for 48 h, then the blowing agent was removed and dried to constant weight in vacuum oven. The content of PVDC was calculated by measuring the content of chlorine element, which was determined by oxygen flask combustion-potentiometric titration method [29]. The content of PAN was measured by determining the content of nitrogen element, which was tested by element analyzer (EA1112, CarloErba, Cornaredo, Italy). The remaining component was the content of PMMA.

The gel content of the microspheres was determined by the extraction method. The weighed microspheres were placed in filter paper bags and extracted by *N, N*-dimethylformamide for 72 h at room temperature, and the *N, N*-dimethylformamide was exchanged for every 12 h. Then the gel was dried to a constant weight by vacuum pumping for 24 h.

Using methanol as a solvent, copolymer microspheres were processed by soxhlet extractor for 48 h to completely remove the blowing agent. Then the *T*_g_ of copolymer microspheres were determined by differential scanning calorimeter (DSC, TA Q200, TA instruments Co., New Castle, DE, USA) at a heating rate of 20 °C/min.

A precision temperature control meter workbench was used at the heating rate of 0.1 °C/s to heat up copolymer microspheres to expand until microspheres collapse or shrink. During this process, the microsphere expansion process was observed with a Leica optical microscope (Leica Camera AG, Solms, Germany), and the microspheres start expansion temperature (*T*_st_), start shrinkage temperature (*T*_sh_), *T*_r_ were determined. *T*_st_ was the temperature at which the copolymer microspheres began to expand after heating. *T*_sh_ was the temperature at which the copolymer microspheres began to crack, shrink, or collapse after heating beyond a certain temperature. *T*_r_ was the temperature range between *T*_st_ and *T*_sh_ of the copolymer microspheres, which indicated the temperature-resistant range of the expanded microspheres. The statistical software was used to calculate the microspheres size of before and after expansion larger than 200 microspheres in the microscopic images, designated as *R*_b_ and *R*_a_, respectively. The *E*_v_ was obtained by the formula of $E_{v}=R_{a}^{3}/R_{b}^{3}$. To accurately determine the quantitative and constant volume of microspheres in a scale test tube, the formula of ρ=mv was used to calculate the apparent density of the microsphere before expansion. The quantitative microsphere sample was fetched in a scale test tube, and placed in an oven and kept at a certain temperature for about 2 min. Then it was taken out and cooled to room temperature and the weight and volume were measured to calculate the apparent density of the microspheres after expansion using the same formula of ρ=mv.

The morphologies of copolymer microspheres before and after expansion were observed on a Carl Zeiss Ultra 55 scanning electron microscope (Carl Zeiss AG, Oberkochen, Germany). Samples were coated with a thin layer of gold with a sputter coater (Quorum/Emitech SC7620, 2 mbar, 240 s at 4–6 mA) before analyzing. Some microspheres were sectioned in order to observe the internal structure.

Taking a set of VDC-MMA-AN copolymer microspheres and the content of blowing agent was determined with TGA method. After that, an electronic scale with a precision of 0.0001 g was used to weigh a group of copolymer microspheres synthesized by different monomers composition with a mass of about 3.5 g (each consisting of three parallel samples) in a Petri dish, paving, placed in a 50 °C oven thermostat, at regular intervals to take out the Petri dishes and weigh resin quality changes. *M*_0_ was the weight of initial time resin and *M*_t_ was the weight loss of polymer microspheres at t moment.

## 3. Results and Discussion

### 3.1. Effects of the Shell Polymer Composition and T_g_ on the Expansion Properties of Polymer Microspheres

**Figure 1:**
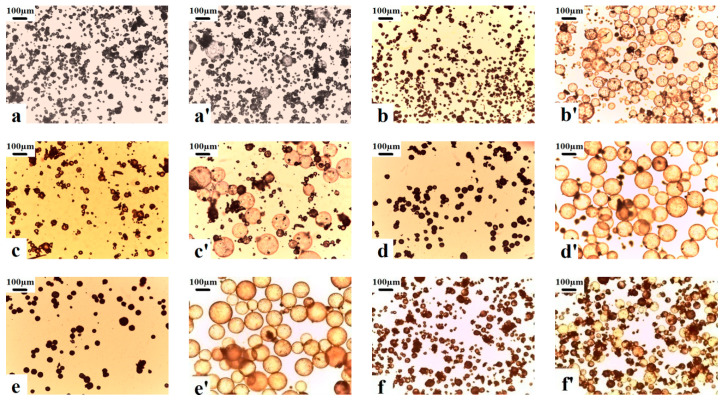
Morphologies of vinylidene chloride-methyl methacrylate-acrylonitrile (VDC-MMA-AN) copolymer microspheres before (**a**–**f**) and after (**a’**–**f’**) expansion prepared at different monomer feeding mass ratio of VDC-MMA-AN: (**a**,**a’**) 60/40/0, (**b**,**b’**) 50/40/10, (**c**,**c’**) 70/20/10, (**d**,**d’**) 50/30/20, (**e**,**e’**) 60/20/20, (**f**,**f’**) 70/10/20.

The *T*_g_ of shell polymer, *T*_st_, *T*_sh_, *T*_r_, and *E*_v_ of copolymer microspheres synthesized by different monomer composition under the condition of pentane as blowing agent and DVB crosslinking agent was 0.2 wt% are shown in Table 1. The microscopic morphologies of the microspheres before and after expansion are shown in Figure 1. Because the reactivity ratios of monomers are different, r_12_ = 0.32, r_21_ = 0.92, r_13_ = 0.36, r_31_ = 2.38, r_23_ = 0.138, r_32_ = 1.322, 1-VDC, 2-AN, 3-MMA [30]. The shell polymer compositions of the vinylidene chloride copolymer microspheres are quite different from that of the actual feeding monomer. The VDC-MMA copolymer microspheres present extremely poor expansion properties. Although there were a few number of the VDC-MMA copolymer microspheres with the feeding mass ratio of 60/40 exhibiting expandable behavior. However, when that microspheres expanded, they easily cracked and collapsed in a narrow *T*_r_, as shown in Figure 1a,a’. It is mainly due to the increase of PVDC segment units in the shell polymer, which decreases the *T*_g_ of shell polymer of the copolymer microsphere to 53.6 °C. While the boiling point of pentane is 36.1 °C. When the expansion temperature exceeded over the boiling point of pentane and *T*_g_ of shell polymer, pentane produced high internal gas pressure inside the microspheres, then the microspheres began to soften and expand at 80 °C. Whereas the microspheres had not yet expanded, they were brittlely cracked and collapsed by the high gas pressure because of the low *T*_g_ of shell polymer. In particular, when the temperature was raised over the *T*_g_ of shell polymer, a higher gas pressure produced by a high blowing agent *E*_r_ (27.2%) in the VDC-MMA copolymer microspheres with the feeding mass ratio of 70/30 resulted in the microspheres bursting and collapsing immediately, and the expansion behavior was almost invisible.

As can be seen from Table 1 and Figure 1, all of the VDC-MMA-AN copolymer microspheres exhibited thermally expandable behavior. Especially, the copolymer microspheres with the monomer feeding mass ratios of VDC-MMA-AN were 50/40/10, 50/30/20, and 60/20/20, respectively. 80% of these copolymer microspheres expanded and exhibited outstanding expansion properties, in which the *T*_r_ were wide and the expanded microspheres remained in perfect spherical shape, as shown in Figure 1b’,d’,e’. However, although the core-shell VDC-MMA-AN copolymer microspheres encapsulated a high blowing agent *E*_r_ (17.2% and 20.2%) with the monomer feeding mass ratio of 70/20/10 and 70/10/20, respectively [27], having the relatively narrow *T*_r_, and some of microspheres cracked or shrank under a slightly higher temperature, therefore they did not keep the uniformity of morphologies and presented a poor expansion properties. Because the chain segment unit of PVDC in the shell polymer increased, the *T*_g_ of shell polymer decreased, the shell polymer was insufficient to resist the internal gas pressure under the high *E*_r_ of blowing agent. Obviously, the monomer composition not only affected the formation of core-shell structure and the *E*_r_ of blowing agent in polymerization process [27], but also impacted the *T*_g_ of shell polymer of copolymer microspheres, and then it further affected the expansion properties of copolymer microspheres.

### 3.2. Effects of the Shell Polymer Crosslinking Degree on the Expansion Properties of Polymer Microspheres

**Figure 2:**
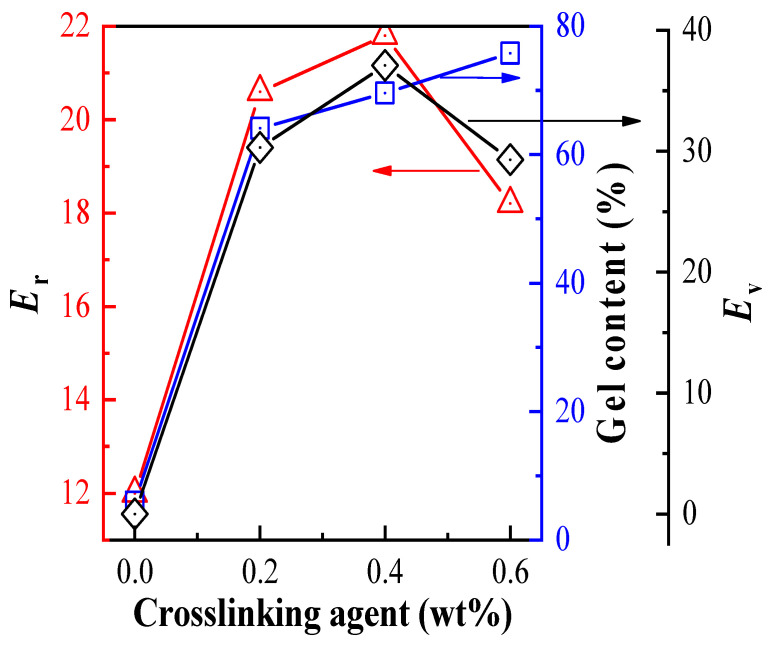
Effects of crosslinking agent content.

**Figure 3:**
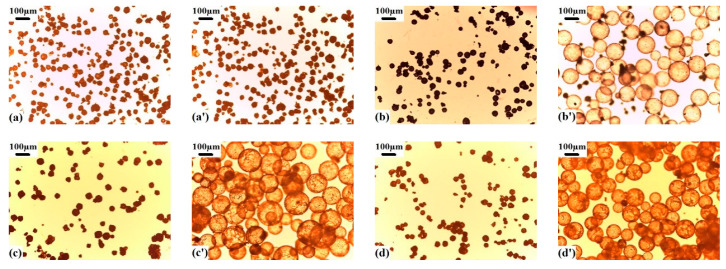
The morphologies of VDC-MMA-AN copolymer microspheres before (**a**–**d**) and after (**a’**–**d’**) expansion with different DVB content: (**a**,**a’**) 0 wt%, (**b**,**b’**) 0.2 wt%, (**c**,**c’**) 0.4 wt%, (**d**,**d’**) 0.6 wt%.

As shown in Table 2, under the fixed monomer feeding mass ratio of VDC-MMA-AN was 50/30/20 and pentane as the blowing agent, the expansion properties of copolymer microspheres with different content of DVB crosslinking agents were significantly different. The impacts of DVB on the gel content in shell polymer, *E*_r_ and *E*_v_ of copolymer microspheres are shown in Figure 2. The morphologies of copolymer microspheres before and after expansion with different DVB content are shown in Figure 3, and the morphologies of copolymer microspheres can be seen in our pre-study [27].

Without DVB crosslinking agent, the microspheres shrank or cracked immediately after expansion. The *T*_st_ is same as the *T*_sh_ as shown in Table 2. The morphologies of microspheres before and after expansion have almost no change as shown in Figure 3a,a’. It was because the *E*_r_ of blowing agent was very low (only 12.0%) and produced relatively low gas pressure. Moreover, because of low shell polymer crosslinking degree, the shell polymer was difficult to resist the internal gas pressure produced by the blowing agent in the expansion process, and the deformation of shell polymer was very poor and easy to crack. As seen from Table 2, when the content of crosslinking agent was increased from 0 wt% to 0.4 wt%, the gel content of shell polymer increased from 5.9% to 69.6%, the *T*_st_ dropped from 80 °C to 75 °C, the *T*_sh_ increased from 80 °C to 105 °C, the *T*_r_ increased from 0 °C to 30 °C, and the *E*_v_ sharply increased from 0 to 37.1. On the one hand, the gas pressure required by the expansion process significantly increased because of the increase of the *E*_r_ of blowing agent. On the other hand, increasing the content of crosslinking agent resulted in the improvement in the crosslinking degree of the shell polymer, then the viscoelasticity properties of the shell polymer enhanced and improved the ability of the shell polymer to resist the gas pressure produced by the blowing agent. However, sequentially increasing the content of DVB to 0.6 wt%, reduced the *T*_st_, *T*_sh_, *T*_r_, and *E*_v_. The gel content of the shell polymer was 75.8% and the crosslinked network of the shell polymer was denser, so that the rigidity of the shell polymer was significantly enhanced, and the ability of the shell polymer to resist deformation was strengthened when the microsphere expanded, even if sufficient internal gas pressure was produced by a higher blowing agent encapsulated content, the microspheres could not exhibit good expansibility.

### 3.3. Influences of the Type and E_r_ of Blowing Agent on the Expansion Properties of Polymer Microspheres

The *T*_st_, *T*_sh_, *T*_r_, and *E*_v_ of copolymer microspheres synthesized by different types of blowing agent when DVB was 0.2 wt% and the monomer feeding mass ratios of VDC-MMA and VDC-MMA-AN were 70/30 and 50/30/20, respectively, as shown in Table 3. Although the *E*_r_ of blowing agent in VDC-MMA copolymer microspheres was 32.1% and 24.2% when using the hexane and heptane as the blowing agent, respectively, the VDC-MMA copolymer microspheres still did not expand, because the boiling point of hexane (68.7 °C) and heptane (99 °C) are higher than the *T*_g_ (53.5 °C) of the shell polymer. When the temperature was raised above the boiling point of hexane and heptane, because of the low *T*_g_ of shell polymer, it resulted in a lower strength and elongation of shell polymer, and the blowing agent leaked and escaped through the broken copolymer shell.

Although the *E*_r_ of blowing agent in the VDC-MMA-AN copolymer microspheres were lower than that of VDC-MMA copolymer microspheres, the VDC-MMA-AN copolymer microspheres with isopentane, pentane, hexane, and heptane as blowing agents showed excellent expansion properties and the *E*_v_ were close to 30, as shown in Table 3. The expandable microspheres-encapsulated isopentane began to expand at 73 °C and shrink at 105 °C, and the *T*_r_ was up to 32 °C. The *T*_st_, *T*_sh_, and *T*_r_ of the microspheres-encapsulated pentane were 76 °C, 100 °C, and 24 °C, respectively. The *T*_st_, *T*_sh_, and *T*_r_ of the microspheres-encapsulated hexane were 85 °C, 100 °C, and 15 °C, respectively. The *T*_st_ and *T*_sh_ of the microspheres-encapsulated heptane was 88 °C and 100 °C, but the *T*_r_ was only 12 °C. Obviously, with the increase of the boiling point of blowing agent, the *T*_st_ gradually increased from 73 °C to 88 °C, while the *T*_r_ decreased from 32 °C to 12 °C.

**Figure 4:**
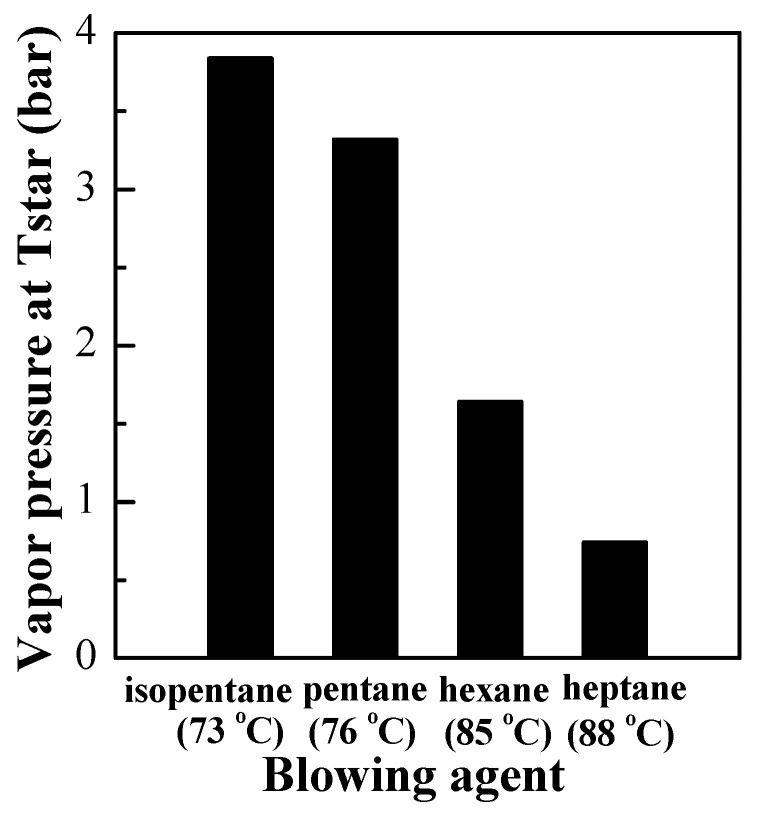
Calculated vapor pressures of different blowing agents at *T*_st_ for microspheres in this study (the temperatures used when calculating the vapor pressures for each blowing agent are given within the brackets).

Therefore, the difference in the boiling point of the blowing agent results in the difference between the expansion properties of microspheres of *T*_st_, *T*_sh_, and *T*_r_, which should seriously affect the application of thermally expandable microspheres. The main reason is that the vapor pressure of the blowing agent at different boiling point is different in the expansion temperature of thermally expandable microspheres. According to literature calculation methods and constants [31], the vapor pressure of the blowing agent at the expansion temperature was calculated, as shown in Table 4. The precondition of this calculation was to assume that all the microspheres were airtight structures and there were no blowing agents diffused through the shell layer and without considering the influence of possible residual monomers. The calculation results are shown in Figure 4. It can be seen that the vapor pressure values of isopentane, pentane, hexane, and heptane at the *T*_st_ are 3.84 bar, 3.32 bar, 1.64 bar, and 0.74 bar respectively. Isopentane could provide high expansion pressure at a lower temperature, therefore the microspheres expanded at a lower temperature.

**Figure 5:**
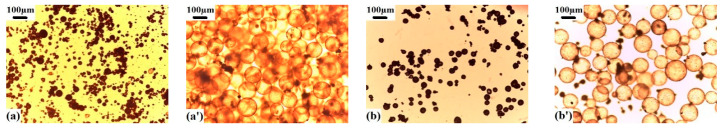

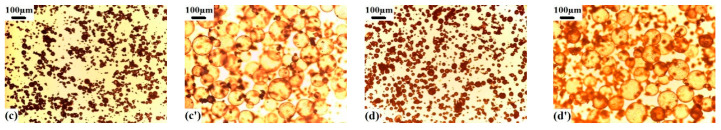
Morphologies of VDC-MMA-AN copolymer microspheres before (**a**–**d**) and after (**a’**–**d’**) expansion: (**a**,**a’**) isopentane, (**b**,**b’**) pentane, (**c**,**c’**) hexane, (**d**,**d’**) heptane.

The morphologies of the VDC-MMA-AN copolymer microspheres before and after expansion are shown in Figure 5. The morphologies of microspheres-encapsulated hexane or heptane were not as smooth as those of pentane, and the surface of microspheres were uneven. The reason was that the microspheres with hexane and heptane as blowing agents had many folds and lots of pits in the polymerization process, which were reported in our pre-study [27]. It could be seen that the type of blowing agent determined the driving force of the expansion process.

**Figure 6:**
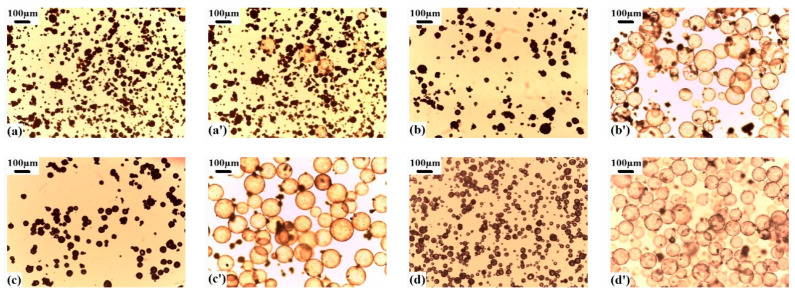
Morphologies of VDC-MMA-AN copolymer microspheres before (**a**–**d**) and after (**a’**–**d’**) expansion with different *E*_r_ of pentane: (**a**,**a’**) 3.4%, (**b**,**b’**) 10%, (**c**,**c’**) 20.6%, (**d**,**d’**) 28.6%.

The parameters of the expansion properties and the morphologies of the VDC-MMA-AN copolymer microspheres with the different content of pentane blowing agents are shown in Table 5 and Figure 6, respectively.

As can be seen in Table 5, increasing the *E*_r_ of pentane, the *T*_st_ and *T*_sh_ gradually decreased, respectively. However, the *T*_r_ was basically maintained at about 24 °C and the *E*_v_ increased from 16.2 to 41.3, the expansion properties of copolymer microspheres were obviously improved. At a lower temperature, the shell polymer was not soft enough and retained high mechanical strength, which required higher vapor internal pressure to expand. However, increasing the *E*_r_ of blowing agent provided copolymer microspheres with high vapor internal gas pressure drove to the microspheres began to expand and shrink at a lower temperature. In addition, with the continuous improvement in the expansion temperature, the mechanical strength of the shell polymer decreased, but the vapor internal pressure produced by the blowing agent continued to increase, especially the higher *E*_r_ of blowing agent was, the greater internal gas pressure produced. Up to the *T*_th_ of the microsphere, the equilibrium between the internal gas pressure and the carrying capacity of the shell polymer to resist the internal gas pressure was destroyed. After that, the internal gas pressure was dominant, and resulted in the shell of microspheres cracking, shrinking, or collapsing. As can be seen from Figure 6a,a’, only a few number of microspheres expanded when the *E*_r_ of pentane was only 3.4%. The blowing agent encapsulated in the microspheres was too low to provide enough expansion gas pressure for the expansion of the microspheres. When increased the *E*_r_ of blowing agent, the numbers of expanded microsphere and *E*_v_ increased significantly, and the surface of the microspheres became round, as shown in Figure 6b’,c’. When the *E*_r_ of pentane continually increased, there was no significant difference in the morphologies of the microspheres, as shown in Figure 6d’. Therefore, from the perspective of expansion properties of microspheres, as long as the *E*_r_ of blowing agent in microspheres is in the range of 10–30%, the microspheres can possess excellent expansion properties.

### 3.4. Expansion Properties of the VDC-MMA-AN Copolymer Thermally Expandable Microspheres

**Figure 7:**
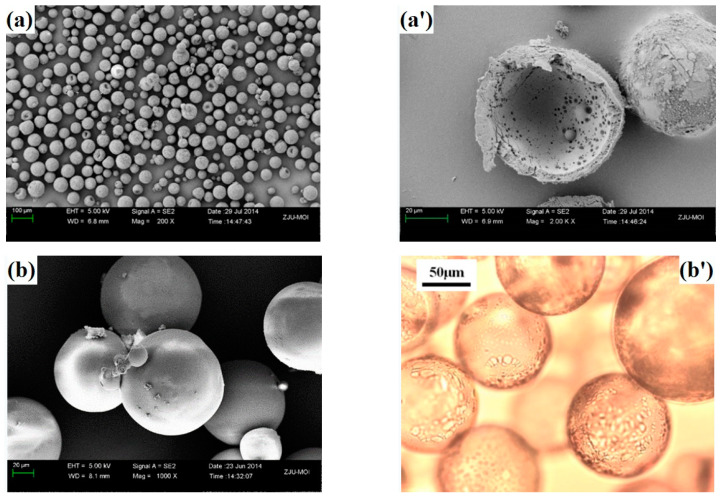
Morphologies of VDC-MMA-AN copolymer microspheres before expansion (**a**,**a’**) and after expansion (**b**,**b’**).

**Figure 8:**
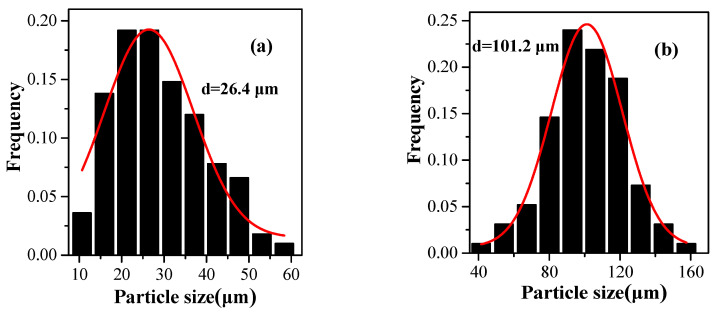
Particle size distribution of VDC-MMA-AN copolymer microspheres before expansion (**a**) and after expansion (**b**).

The typical VDC-MMA-AN copolymer thermally expandable microspheres were synthesized by 0.2 wt% of DVB, 28.6 wt% of pentane as blowing agent and the monomer feeding mass ratio of VDC-MMA-AN was 50/30/20. The morphologies and particle size distribution before and after expansion of the VDC-MMA-AN copolymer microspheres are shown in Figure 7 and Figure 8, respectively. All the copolymer microspheres are core-shell structure, and the thickness of shell layer are uniform, as shown in Figure 7a,a’, and the average size of copolymer microspheres is about 26.4 μm (shown as Figure 8a). After the expansion, the *E*_v_ of the microspheres are more than 41.3 times as mentioned above. The expanded microspheres are round with the smooth surface (shown as Figure 7b,b’), and the average size of expanded microspheres is about 101.2 μm (shown as Figure 8b). Moreover, the apparent density of copolymer microspheres dramatically reduced from about 0.4–0.5 kg·m^−3^ before expansion to 0.0125 kg·m^−3^ after expansion, decreasing over than 40 times. The VDC-MMA-AN copolymer microspheres exhibited excellent expansion properties.

### 3.5. Diffusion of Blowing Agent in the VDC-MMA-AN Copolymer Thermally Expandable Microspheres

**Figure 9:**
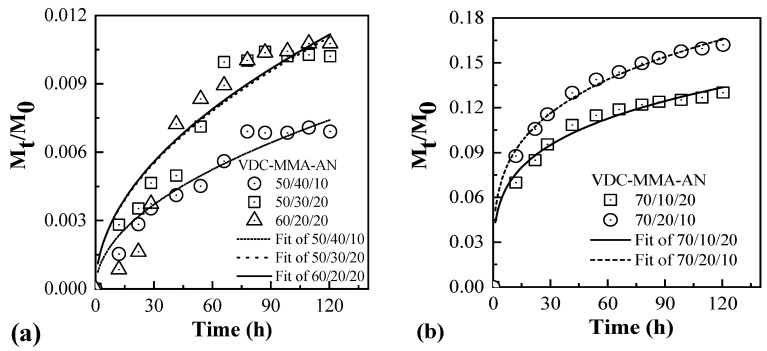
Diffusion behavior of pentane in VDC-MMA-AN copolymer microspheres with different compositions at 50 °C.

VDC-MMA-AN copolymer microspheres were selected as the research objects, and the various loss of weight measurements of the polymer microspheres were recorded in a certain time at 50 °C. As shown in Figure 9a, the loss weight of blowing agent was less than 0.5 wt% at the first 30 h in the VDC-MMA-AN copolymer microspheres, and the feeding mass ratios of VDC-MMA-AN were 50/40/10, 50/30/20, and 60/20/20, respectively. It satisfied the formula of $M_{t}/M_{0}=kt^{n}$ and the fitting parameters are shown in Table 6. It can be seen that it fitted Fick diffusion when *n* = 0.5, which belongs to the general diffusion behavior. The results show that the copolymer microspheres at such feeding mass ratio possessed excellent barrier properties and retention to pentane blowing agent. However, when the feeding mass ratios of VDC-MMA-AN were 70/20/10 and 70/10/20, the loss of weight of blowing agent in copolymer microspheres was nearly 1.0 wt% at the first 30 h, and then the loss rate slowly decreased, as shown in Figure 9b. By fitting the experimental data, it belonged to non-Fick diffusion when ½ < *n* < 1, and the fitting parameters *k* and *n* values are shown in Table 6 [33,34].

When the feeding mass ratios of VDC-MMA-AN were 50/40/10, 50/30/20, and 60/20/20, the *T*_g_ of shell copolymer of copolymer microspheres were 63.8 °C, 63.4 °C, and 61.5 °C, respectively, which were all higher than the experimental temperature (50 °C). Therefore, under the 50 °C test conditions, the shell copolymers were in a glass state resulting in the low activity of the molecular chain of shell copolymer, the solubility and diffusion velocity of blowing agent in shell polymer were low, in which the diffusion process approached to the Fick diffusion. However, the *T*_g_ of shell copolymer of VDC-MMA-AN copolymer microspheres with the feeding mass ratios of 70/20/10 and 70/10/20 were 51.4 °C and 42.4 °C, respectively, which were close or below to the experimental temperature (50 °C). Therefore, the shell copolymer in such test condition was in the glass transition zone or the high elastic zone, resulting in an increase in the molecular chain activity of shell copolymer. The solubility and diffusion velocity of blowing agent in shell polymer increased. In order to ensure a favorable expansion effect of low-temperature thermally expandable microspheres in the future application process, it should be preserved at a lower temperature.

### 3.6. Thermally Expandable Microspheres Designation

From the previous experimental results, in order to prepare microspheres with excellent expansion properties, both of the properties of shell polymer and blowing agent must be considered. selection of gas-proof shell polymeric composition, simultaneous, balance of *T*_g_, and melt viscosity of shell polymer, types and boiling point of blowing agent must be important. Further, when exposed to heat, ductility and intensity of shell polymer also must be an important factor [21,27]. The properties of shell polymer are mainly displayed by the viscoelasticity, *T*_g_, crosslinking degree and compatibility with blowing agent, etc. Shell polymer properties are mainly controlled by shell composition and crosslinking degree. The molecular chain structure, the polymer modulus, and mechanical strength of high elastic states are closely related to the entanglement density and crosslinking degree of the molecular chain of the shell polymer. By adding crosslinking agent, the crosslinked network structure is an effective method to improve the high elastic modulus, elasticity, mechanical strength, and temperature range of high elastic state. However, when the content of crosslinking agent is too high, the crosslinking density and elastic resilience of the molecular chain would increase, which may restrict the expansion process. The adjustment of *T*_g_ can be achieved by the copolymerization of hard monomer and soft monomer. For copolymer microspheres containing VDC monomer, it is necessary to add sufficient contents of co-monomer to destroy the crystallinity of PVDC homopolymer. Then the *T*_g_ range of copolymer can be controlled by adjusting monomer composition. On the other hand, the expansion of polymer microspheres requires a driving force, which is the internal gas pressure is produced by the vaporization of the blowing agent. Therefore, the blowing agent structure determines the boiling point and the vapor pressure at a certain temperature. The *T*_st_ can be adjusted by the combination of the content of the blowing agent with the appropriate boiling point. The boiling point of the blowing agent is lower than the softening temperature of the shell polymer, and it has enough internal gas pressure at the expansion temperature to drive the expansion of the microspheres.

## 4. Conclusions

The compatibility among the shell polymer composition, *T*_g_, and the boiling point of blowing agent is a key factor in affecting the expansion properties of vinylidene chloride copolymer microspheres. VDC-MMA-AN copolymer microspheres demonstrated excellent expansion properties, but it was difficult to expand for VDC-MMA copolymer microspheres. The *E*_v_ and *T*_r_ were improved by adding 0.2–0.4 wt% of DVB. The *T*_g_ of the shell polymer must be higher than the boiling point of the blowing agent. The lower the boiling point of the blowing agent, the higher the internal gas pressure driven microsphere expansion, and the wider the *T*_r_. While increasing the *E*_r_ of blowing agent improved the *E*_v_ of the microspheres. The *T*_st_ and *T*_r_ were adjusted by modifying the type and *E*_r_ of the blowing agent.

The VDC-MMA-AN thermally expandable microspheres had excellent expansion properties, in which the *E*_v_ was more than 41 times, and the apparent density dramatically reduced from about 0.4–0.5 kg·m^−3^ to 0.0125 kg·m^−3^ after expansion, beyond more than 40 times.

Pentane in the VDC-MMA-AN copolymer microspheres prepared with the VDC-MMA-AN feeding mass ratios of 50/40/10, 50/30/20, and 60/20/20 were in the form of Fick diffusion, the blowing agent had a good retention rate. However, the diffusion of pentane blowing agent in the VDC-MMA-AN copolymer microspheres with the feeding mass ratios of 70/20/10 and 70/10/20 basically followed the non-Fick diffusion mechanism, and the loss of blowing agent was very fast. Low-temperature thermally expandable microspheres should be preserved at a lower temperature.

## Figures and Tables

**Table 1:** Expansion characteristics of polymer microspheres synthesized at different monomer feeding mass ratio.

VDC/MMA/AN (Mass)	Shell Composition of PVDC/PMMA/PAN	*E*_r_ (%)	*T*_g_ (^o^C)	*T*_st_ (^o^C)	*T*_sh_ (^o^C)	*T*_r_ (^o^C)	*E*_v_
50/50/0	46.1/53.9/0	1.7	56.3	-	-	-	0
60/40/0	54.6/45.4/0	6.8	53.6	80	85	5	3
70/30/0	61.0/39.0/0	27.2	53.5	-	-	-	0
50/40/10	48.5/42.0/9.5	17.5	63.8	80	105	25	19
70/20/10	61.5/29.3/9.2	17.2	51.4	55	65	10	7.9
50/30/20	41.2/41.0/17.8	20.6	63.4	76	100	24	30.3
60/20/20	48.8/31.5/19.7	20.4	61.5	70	90	20	28.9
70/10/20	61.7/17.9/20.4	20.2	42.4	65	75	10	27

**Table 2:** Expansion properties of copolymer microspheres with the different shell polymer crosslinking degree.

DVB (wt%)	Gel Content (%)	*E*_r_ (%)	*T*_st_ (^o^C)	*T*_sh_ (^o^C)	*T*_r_ (^o^C)	*E*_v_
0	5.9	12.0	80	80	0	0
0.2	64.1	20.6	76	100	24	30.3
0.4	69.6	21.8	75	105	30	37.1
0.6	75.8	18.2	69	95	27	29.3

**Table 3:** Expansion characteristics of copolymer microspheres synthesized at different type of blowing agent.

VDC-MMA-AN (Mass)	Blowing Agent	*E*_r_ (%)	*T*_st_ (^o^C)	*T*_sh_ (^o^C)	*T*_r_ (^o^C)	*E*_v_
70/30/0	pentane	27.2	-	-	-	0
70/30/0	hexane	32.1	-	-	-	0
70/30/0	heptane	24.2	-	-	-	0
50/30/20	isopentane	19.8	73	105	32	28.6
50/30/20	pentane	20.6	76	100	24	30.3
50/30/20	hexane	21.0	85	100	15	29.8
50/30/20	heptane	22.2	88	100	12	28.2

**Table 4:** Boiling points and data used for calculating Vapor Pressures * of the hydrocarbons used as blowing agents in this study [24,32].

Blowing Agent	Boiling Point (°C)	Solubilitypara Meter (Cal/cm^3^)^1/2^	Vapor Pressure Constants				
			C1	C2	C3	C4	C5
isopentane	28	6.7	72.350	−5010.9	−7.8830	8.9795 × 10^−6^	2
pentane	36.1	7.0	78.741	−5420.3	−8.8253	9.6171 × 10^−6^	2
hexane	68.7	7.3	104.650	−6995.5	−12.7020	1.2381 × 10^−5^	2
heptane	99	7.4	87.829	−6996.4	−9.8802	7.2099 × 10^−6^	2

* Vapor pressure (in bar) = exp [C1 + (C2/T) + C3 × ln (T) + C4 × T^C5^] × 10^−5^; temperatures are in Kelvin.

**Table 5:** Expansion characteristics of copolymer microspheres with different *E*_r_ of pentane.

*E*_r_ (%) of Pentane	*T*_st_ (^o^C)	*T*_sh_ (^o^C)	*T*_r_ (^o^C)	*E*_v_
3.4	87	110	23	16.2
10.0	80	105	25	32.2
20.6	76	100	24	30.3
28.6	70	93	23	41.3

**Table 6:** Diffusion mechanism of pentane in VDC-MMA-AN copolymer microspheres.

VDC-MMA-AN	*t*		*n*		Adj. R-Square
	Value	Standard Error	Value	Standard Error	
50/40/10	6.76 × 10^−4^	1.87 × 10^−5^	0.50	2.51 × 10^−7^	0.93
50/30/20	1.01 × 10^−3^	3.93 × 10^−5^	0.50	2.10 × 10^−7^	0.88
60/20/20	1.02 × 10^−3^	5.75 × 10^−5^	0.50	2.37 × 10^−7^	0.83
70/10/20	0.041	0.0032	0.25	0.018	0.96
70/20/10	0.049	0.0022	0.26	0.011	0.99
